# Supplementary figures and images for: Titer estimation for quality control (TEQC) method: A practical approach for optimal production of protein complexes using the baculovirus expression vector system
Source: PLoS One. 2018 Apr 3;13(4):e0195356. doi: 10.1371/journal.pone.0195356 (PMC5882171; doi:10.1371/journal.pone.0195356)

|                                            |      |      |      |      |      |
|--------------------------------------------|------|------|------|------|------|
| Virus volume from original stock (ml)      | 0.02 | 0.04 | 0.06 | 0.08 | 0.1  |
| Cell density ( $1.0 \times 10^6$ cells/ml) | 2.25 | 1.82 | 1.62 | 1.46 | 1.37 |
| eMOI                                       | 0.13 | 0.56 | 0.84 | 1.13 | 1.35 |
| IU (Infectious Units ( $10^7$ ))           | 0.67 | 2.78 | 4.18 | 5.67 | 6.76 |

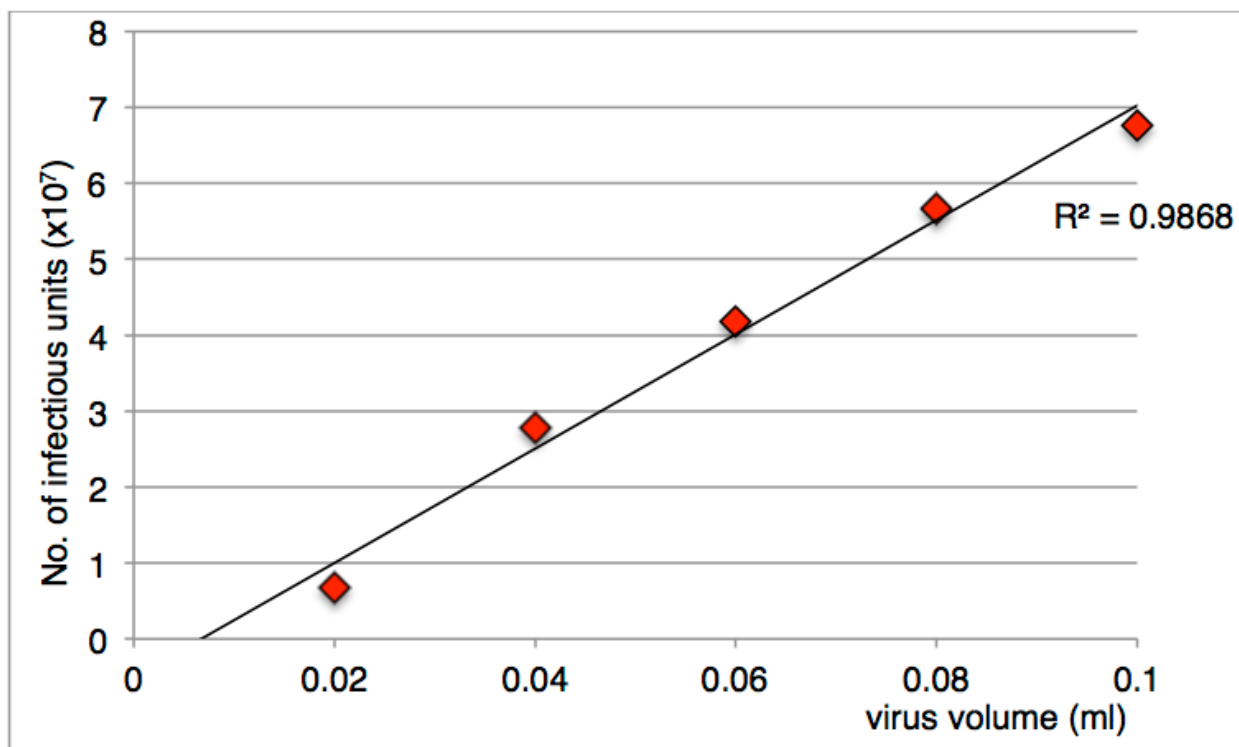

Supplement: S7 Fig — Cell density at each virus volume was measured, and its corresponding eMOI/eTiter were calculated as summarized on top. Linear regression is derived from 5 different measurement points and plotted in bottom. X-axis: virus volume (ml); Y-axis: number of infectious units. (PDF) [file pone.0195356.s007.pdf]
