# Supplementary material for: Titer estimation for quality control (TEQC) method: A practical approach for optimal production of protein complexes using the baculovirus expression vector system
Source: PLoS One. 2018 Apr 3;13(4):e0195356. doi: 10.1371/journal.pone.0195356 (PMC5882171; doi:10.1371/journal.pone.0195356)
Supplement: S1 File — (PDF) [file pone.0195356.s008.pdf]

S1 File:

Supplemental Protocol

Estimation of Titer/MOI

Overview:

Our work has demonstrated that the optimized expression of multi-protein complexes in insect cells lies in controlling the level of virus infection. Our TEQC method is simple in that it only requires measuring cell density of the insect cells 24 hours after addition of recombinant baculovirus. Titer can be easily estimated from the equation: (eMOI × initial cell density) / volume of virus added. Expression of protein complexes can be described as a function of eMOI, enabling optimization and consistent expression of protein complexes in the insect cells using the baculovirus expression vector system (BEVS). The detailed description of the protocol is as follows:

(I) General cell culture reagents and equipment:

Reagents we use for cell culture and protein production are as follows:

- ESF921 medium (Expression systems)

- Transfection Medium (Expression systems)

- Sf9 cells or Hi5 cells (Expression systems)

- Cellfectin II (Life Technologies)

- *E. coli* competent cells for general cloning (such as XL1Blue, DH10α)

- *E. coli* competent cells lack R6Kγ replication origin (PirPlus, BW23473)

- *E. coli* competent cells DH10MultiBac [4]

- Vector DNA (pFL, pKL, pUCDM, pSPL) [4]

- 250 ml (Corning Erlenmeyer vent cap 250ml)

- 1.8 ml cryo tubes

- freezing container (Nalgene)

- Shaker
- Culture room maintained at 27 °C
- Microscope
- Hemocytometer or cell counter
- Centrifuge (Thermo Fisher Centra CL2 centrifuge)
- BSA (Chem-Impex)
- DMSO (Thermo Fisher)

(II) Virus production

The maintenance of the insect cells as well as amplification of baculoviruses is carried out using a shaker with disposable culture flasks. We follow the protocol for virus production described in [4].

(III) Virus storage

A virus titer declines over a period of time if kept at 4 °C. Such change of virus titer will effect the outcome of protein productions in the insect cells [5]. In order to maintain the consistency of protein production, we have implemented the TIPS method to generate frozen stocks [6]. The key point of this protocol is that a baculovirus is frozen not in a liquid but in the form of 'infected cells' - the cells serves as cryo-protectant. The step-by-step procedure is described below:

1. Generate V1 baculovirus by following the protocol described in Fitzgerald et al [4].

2. Prepare 200 ml of Sf9 cells containing 0.4×10^6 cells/ml in a 1L flask

3. Infect cells with a V1 virus by adding 0.1 ml of V1 virus to the culture. Based on our experience, eMOI should be less than 0.1 to prevent defective virus production. For a rough estimate, if eMOI is 1.0, the cell number will be around 0.6×10^6 cells/ml, 24 hour after

infection (see more detail at section IV).

4. Count and split cells every 24 hours in order to maintain cell number at less than 1.0×10^6 cells/ml - this is very important - until cell proliferation arrests - at this stage, the morphology of the cells clearly indicates a state of full infection (best between 0.5×10^6 - 0.7×10^6 cells/ml). When a growth proliferation arrest is observed, the virus-infected cells should be harvested (see below).

5. Prepare cryo-protection buffer (100ml):

- 90 ml ESF921 medium (Expression Systems)
- 10 g/L of BSA (Chem-Impex)
- 10 % DMSO (Thermo Fisher)

Mix all reagents well and filter sterilize.

6. Transfer the culture into 50 ml conical tubes, centrifuge for 5 min with 1,500 rpm at RT using Centra CL2 centrifuge (Thermo Fisher), and discard supernatant.

7. Resuspend cells gently with cryo-protection buffer to the final cell density of 10.0×10^6 cells/ml. For example, if the final cell density was 0.5×10^6 cells/ml in a 200 ml culture, then the cell pellet contains 100×10^6 cells/ml, and thus, 10 ml cryo-protection buffer should be used to resuspend the cells.

8. Make 1ml aliquots in cryo tubes and immediately place them in a freezing container (Nalgene) at -80 °C for 24 hours followed by storage in liquid nitrogen. Frozen stocks should be ready for use after 2 full days equilibration in liquid nitrogen. Note: Typically, a titer of frozen stock is about 8-10 times higher than that of an original V1 virus.

(IV) Determination of I_{24}, eMOI, and eTiter

Using our formula (Eqs. 1, 4, 5), I_{24}, eMOI, Infectious units (IU), and eTiter can theoretically be calculated given the volume of baculovirus added to the insect cell culture, and cell density at 24

hours after addition of virus. In practice, however, I_{24} at MOI greater than 2 (MOI>2) is nearing saturation, for example, I_{24}=86% (MOI=2), and I_{24}=95% (MOI=3). Therefore, a range of virus volumes, corresponding to an eMOI value of between 0.1 and 2.0, semi-linear range, should be used. Moreover, to minimize overall error. For accuracy, a cell growth constant, α, should be measured at the same time when I_{24} is measured under the same condition. eMOI can be delineated by linear regression as described below:

IU (eMOI × initial total cell number) = 'eTiter' (slope) × virus volume

The details of the procedure for eTiter and eMOI determination are described below.

We use 1.0×10^6 cells/ml for Hi5 cells and 1.5×10^6 cells/ml for Sf9 cells: C_0

1. Prepare 50 ml of Hi5 cells in 250 ml flask containing 1.0×10^6 cells/ml, or Sf9 cells containing 1.5×10^6 cells/ml.
2. Thaw the frozen virus stock as quickly as possible - usually warming it up by holding it in your palm, or use liquid virus stored at 4 °C.
3. Prepare five-fold serial dilutions of virus solution. Place 5 sterile 1.5 ml tubes (or 15 ml tube for liquid virus), add 800 µl (or 8 ml for liquid virus) of medium, and label them "1", "2", "3", "4", and "5". Make dilution series by adding 200 µl of thawed virus frozen stock (or 2 ml liquid virus) to the tube "1". Mix virus frozen stock thoroughly by pipetting up and down before dilution. Close the cap of the "1" tube and invert to mix well. Take 200 µl (or 2 ml for liquid virus) of the "1" to the tube "2". Continue this process to the tube "5".
4. Infect cells with 0.5 ml (or 5 ml for liquid virus) of series of the diluted virus solutions by pipetting 0.5 ml (or 5 ml for liquid virus) from the tubes #1, 2, 3, 4, 5, and adding each into each 50 ml culture. In addition, set up one additional 50 ml culture with no virus added, which will be used to measure α value. Incubate them on shaker at 100 rpm, 27°C for 24 hours.
5. 24 hours post infection, count cell number using a hemocytometer, or cell counter to determine cell density: C_1. If I_{24} is around 10% to 90%, the cell number should be between 1.2-2.0 × 10^6 cells/ml in Hi5 culture.

6. Use the following equation to calculate infectivity and eMOI for each virus dilution.

$$I_{24}\,estimate(eI_{24}) = 1 - \frac{C_1 - C_0}{C_0(\alpha - 1)}$$

$$eMOI = -\ln(1 - eI_{24}) = -\ln\left(\frac{C_1 - C_0}{(\alpha - 1)C_0}\right)$$

C_0: initial cell density

C_1: cell density 24 hours after infection

α: Growth constant indicates fold change of uninfected cells in a 24 hours period measured under the same condition used for protein expression. In our case, α is 2.43 for Hi5 and 2.19 for Sf9 cells respectively.

Note: The α value should be experimentally determined using the insect cell lines used for expression of a protein of your interest.

7. Use the following equation to calculate eMOI, with which number of infectious units (IU) in 50 ml culture can be calculated:

$$IU = eMOI \times C_0\ (cells/ml) \times culture\ volume\ (ml)$$

As IU = eTiter × virus volume, 'eTiter' can be described as 'slope' of a linear plot with virus volume being X-value and IU being Y-value:

$$IU\ (Y\text{-value}) = \text{'eTiter'}\ (slope) \times virus\ volume\ (X\text{-value})$$

Given multiple data points of IU and virus volume, eTiter can be delineated by a linear regression.

For example, in the case of the Mediator Head module, we determined eMOI and IU by infecting cells with serial dilutions of 1/5 diluted virus and measured C_1 values with each dilution. The data are summarized in S2 Table.

Plot the IU at Y-axis vs. virus volume at X-axis. Use a linear regression to derive a formula for IU as a function of virus volume (**S7 Fig**).

$$\text{eTiter} = 1.506 \times 10^{8} \text{ (IU/ml)}$$

What this number indicates is that 1 ml of the Head module-expressing virus contains a total of 1.506×10^{8} infectious units; when 1 ml of the virus is added to 50 ml Hi5 cell culture with cell density of 1.0×10^{6} cells/ml (total 5.0×10^{7} cells in the culture), eMOI will give rise to 1.506×10^{8} infectious units / 5.0×10^{7} cells = 3.0 (IU/cell).

References:

1. Imasaki T, Calero G, Cai G, Tsai KL, Yamada K, et al. Architecture of the Mediator head module. Nature 2011; 475:240-243

2. Bieniossek C, Papai G, Schaffitzel C, Garzoni F, Chaillet M, et al. The architecture of human general transcription factor TFIID core complex. Nature 2013; 493:699-702

3. Fitzgerald, DJ, Schaffitzel C, Berger P, Wellinger R, Bieniossek C, Richmond, T.J., and Berger, I. Multiprotein expression strategy for structural biology of eukaryotic complexes. Structure 2007; 15:275-279.

4. Fitzgerald DJ, Berger P., Schaffitzel C, Yamada K, Richmond TJ et al. Protein complex expression by using multigene baculoviral vectors. Nat. Methods 2006; 3:1021-1032.

5. Jorio H, Tran R, Kamen A. Stability of serum-free and purified baculovirus stocks under various storage conditions. Biotechnology progress 2006; 22:319-325.

6. Wasilko DJ, Lee SE, Stutzman-Engwall KJ, Reitz BA, Emmons TL, et al. The titerless infected-cells preservation and scale-up (TIPS) method for large-scale production of NO-sensitive human soluble guanylate cyclase (sGC) from insect cells infected with recombinant baculovirus. Protein Expr. Purif. 2009; 65:122-132.
