# Supplementary material for: Titer estimation for quality control (TEQC) method: A practical approach for optimal production of protein complexes using the baculovirus expression vector system
Source: PLoS One. 2018 Apr 3;13(4):e0195356. doi: 10.1371/journal.pone.0195356 (PMC5882171; doi:10.1371/journal.pone.0195356)
Supplement: S1 Table — (PDF) [file pone.0195356.s009.pdf]

**S1 Table.** Plasmids for complex expression used in this study

| Name | Description | Source |
|---|---|---|
| pYT115 | pSPL-Med20- Med18Δ109-140 | (Imasaki et al., 2011) |
| pYT120 | pSPL- Med6-Med22-Med11-Med8 | (Imasaki et al., 2011) |
| pYT171 | pFL-10His-Med17(delN108) | (Imasaki et al., 2011) |
| Mediator Head module (pYT323) | pYT115+pYT120+pYT171 | (Imasaki et al., 2011) |
| TI542 | pFL+pUCDM | In this study |
| TI543 | pFL+pUCDM+pSPL | In this study |
| Taf8-Taf10 | pPBac-810 | (Bienioseek et al., 2013) |
| pYT444 | pSPL-Tfb2-Ssl1 | In this study |
| pYT465 | pUCDM-Tfb1His-Tfb4 | In this study |
| TI132 | pFL-Tfb5-Rad3 | In this study |
| TFIIHcore (TI419) | pYT444+pYT465+TI132 | In this study |
| pYT154 | pUCDM-Tfg3 | In this study |
| TI150 | pFL-Tfg1_Flag_TAP-Tfg2 | In this study |
| TFIIF (TI153) | pYT154+ pFL-Tfg1_Flag_TAP-Tfg2 | In this study |
| pYT379 | pUCDM-CDK8-CycC | In this study |
| CDK8-CycC (TI600) | pFL+pYT379 | In this study |
| pYT53 | pFL-Med7-Med21 | In this study |
| pYT55 | pFL-Med10-Med31HA-His | In this study |
| pYT56 | pFL-Med9-Med4 | In this study |
| pYT57 | pFL-Med19-Med1 | In this study |
| pYT85 | pFL-Med10-Med19-Med1-Med31HA-His | In this study |
| pYT87 | pFL-Med4-Med7-Med21-Med9 | In this study |
| Mediator middle module (pYT90) | pFL-Med4-Med7-Med10-Med19-Med1-Med31HA-His -Med21-Med9 | In this study |
